# Healthcare Providers' Perspective about the Use of Telemedicine in Egypt: A National Survey

**DOI:** 10.1155/2022/3811068

**Published:** 2022-03-12

**Authors:** Mohamed Alboraie, Mohammad Abdalgaber, Naglaa Youssef, Inas Moaz, Nermeen Abdeen, Hazem Maarouf Abosheaishaa, Mina Tharwat Shokry, Fathiya El-Raey, Sabry Shaaban Asfour, Waleed A. Abdeldayem, Adel Ahmed Hassan, Essam Eldeen M. o. Mahran, Mohammed Tag-Adeen, Omar Elshaarawy, Mohamed Ibrahim Radwan, Ahmed Altonbary, Yasser Fouad

**Affiliations:** ^1^Department of Internal Medicine, Al-Azhar University, Cairo, Egypt; ^2^Gastroenterology Department, Police Authority Hospital, Egypt; ^3^Medical-Surgical Nursing Department, Faculty of Nursing, Cairo University, Giza, Egypt; ^4^Medical-Surgical Nursing Department, College of Nursing, Princess Nourah Bint Abdulrahman University, Riyadh, Saudi Arabia; ^5^Epidemiology and Preventive Medicine, National Liver Institute, Menoufia University, Menoufia, Egypt; ^6^Tropical Medicine, Faculty of Medicine, Alexandria University, Alexandria, Egypt; ^7^Egyptian Ministry of Health, Cairo, Egypt; ^8^Department of Tropical Medicine and Gastroenterology, Faculty of Medicine, Aswan University, Aswan, Egypt; ^9^Department of Hepatogastroenterology and Infectious Diseases, Al-Azhar University, Damietta, Egypt; ^10^Matrouh General Hospital, Matrouh, Egypt; ^11^Department of Tropical Medicine, Zagazig University, Zagazig, Egypt; ^12^Infectious and Endemic Disease Department, Suez Canal University, Ismailia, Egypt; ^13^Department of Tropical Medicine and Gastroenterology, Faculty of Medicine, Assiut University, Assiut, Egypt; ^14^Department of Internal Medicine, Qena Faculty of Medicine, South Valley University, Qena, Egypt; ^15^Hepatology and Gastroenterology Department, National Liver Institute, Menoufia University, Egypt; ^16^Department of Gastroenterology and Hepatology, Mansoura University, Mansoura, Egypt; ^17^Endemic Medicine Department, Minia University, Minia, Egypt

## Abstract

Incorporation of telemedicine in general clinical practice is becoming a compelling need nowadays in the context of COVID-19 pandemic and its consequent burdens on the healthcare systems. Though telemedicine appears to be appealing and carries a lot of advantages, yet it is still faced by many challenges and barriers especially in developing countries. Our aim was to explore the impression of healthcare providers about telemedicine and its applicability in clinical practice in Egypt. A cross-sectional study was conducted among healthcare providers from different Egyptian governorates through a web-based survey. The survey gathered information about demographic, socioeconomic features of the enrolled healthcare participants; their knowledge, previous experience, impression about telemedicine, advantages of telemedicine over traditional medical services, barriers that may face telemedicine, and additional services that can be provided by telemedicine were also explored. Our study enrolled 642 healthcare providers from all over Egypt, 43.77% were females, of which 55.5% were physicians, 27.3% were nurses, 6.1% were technicians, 7.6% were administrative clerks, and 3.6% were medical directors. Sixty-four percent of participants reported that they have never used telemedicine. Smartphones were the most commonly used mean in the group who used telemedicine (65%), and smartphone applications were the favorable telemedicine service for about 50% of participants. Participants assumed that the use of telemedicine might not have a negative effect on the doctor-patient relationship but raised some concerns regarding the privacy and security of patients' data. Despite the fact that telemedicine appears to be appealing and widely accepted by healthcare providers, yet still, its implementation is confronted by some obstacles. Precise organizational guidelines need to be developed to clearly figure out the exact role of each healthcare provider to minimize their doubtfulness about telemedicine and to facilitate its adoption.

## 1. Introduction

Telemedicine refers to providing different clinical services to patients at a distance as defined by the World Health Organization (WHO). Historically, it has been dated to the 19th century during the civil war where soldiers used to communicate with their doctors via telegraphs and phones [1]. With the recent advancements and the wide availability and utilization of information computer technologies (ICTs), telemedicine has witnessed great breakthroughs and developments, rapidly creating a new environment for healthcare service and delivery [2]. Replacement of the traditional face to face communication methods with the computer-based ones, hand in hand with the fast decline in the costs of ICTs, has empowered different healthcare institutions to adopt novel and structured methods of presenting care to patients. Moreover, the accessibility and popularity of the Internet nowadays has hastened the tempo of ICT progress, thereby broadening the horizon of telemedicine to include different platforms and online applications (e.g., e-mail messages, video call consultations, and online conferences) as well as interactive media facilities such as digital imaging and videos [3]. There are two main forms of telemedicine sorted according to the timing of data transmission and interactions between the involved personnel; it may be between two doctors or between a doctor and a patient [2]. The first form is named asynchronous telemedicine, which involves the exchange of prerecorded information between two or more persons present at different times for example an email or a smartphone application messages. This is in contrast to real-time or synchronous telemedicine which necessitates the simultaneous presence of the two involved persons at the same time for concurrent data exchange as in case of video calls [4]. In both synchronous and asynchronous telemedicine, relevant data can be exchanged via different forms, such as text message, audio notes, videos, or photos. These two basic telemedicine forms have been applied successfully in many domains and in relation to diverse clinical settings such as teleuroloy, telepsychiatry, telepathology, teledermatology, and teleradiology [5–7]. Incorporation of telemedicine in general clinical practice is becoming a compelling need nowadays in the context of COVID-19 pandemic, and its consequent burdens on the healthcare systems, WHO, and Center of Disease Control and Prevention (CDC) recommend the utilization of telemedicine for patient monitoring, triaging, consultations, and follow-up to minimize the undue pressures on the different healthcare facilities, saving times and efforts of both doctors and patient as well as implementing the recommended social distancing to face this pandemic [6]. Though telemedicine appears to be appealing and carries a lot of advantages, yet it is still faced by many challenges and barriers especially in developing countries, such as the lack of infrastructures in most of healthcare institutions, the deficient awareness and skills of many people in using technology, the geographical and cultural barriers especially in rural areas and the high costs of implementing such services in all hospitals [8]. So, we conducted this study to assess the usefulness of telemedicine application and the different barriers hindering its utilization in our community.

## 2. Methods

A cross-sectional study using a web-based survey was conducted on participants from different Egyptian healthcare facilities. Participants included physicians, nurses, technicians, administrative clerks, and hospital managers. The preparation of the present manuscript run in compliance with the recommendations of the Strengthening the Reporting of Observational Studies in Epidemiology (STROBE) statement; all procedures were done after ethical approval from participating centers. An Arabic survey was designed to evaluate experience of health system staff about telemedicine in their clinical practice. Corresponding participants in the study distributed the survey to at least 40 healthcare workers in their institutes (*n* = 36) all over the 27 governorates of Egypt (total invitations = 1080), and the survey was filled in an individual manner after signing an informed consent to participate in the study. The survey was composed of two sections with multiple choice questions; Section 1 describes the demographic and socioeconomic features of the enrolled healthcare participants.  Section 2 collected data about knowledge of telemedicine by healthcare providers. This section was divided into 4 parts: The first part collected data about previous experience of telemedicine services in clinical practice, the most preferable technological devices and applications to perform telemedicine service. The second part collected data about impression of healthcare workers of telemedicine services over the traditional medical services. This part included 4 domains: The first and second domains assessed advantages of telemedicine services, and the third domain assessed disadvantages of telemedicine services. Each question of the first, second, and third domains was answered by a 5-grade scale of strongly disagree, disagree, equivocal, agree, and strongly agree. The fourth domain screened the participants' opinion about preference of telemedicine services over the traditional medical services using a 10-grade scale of not preferred at all up to mostly preferred. The third part shows screened barriers that may face telemedicine, and the fourth part screened the participants' opinion in the additional services that can be provided for healthcare providers by telemedicine. All items in the third and fourth parts were answered be model of a 5-grade scale of strongly disagree, disagree, equivocal, agree, and strongly agree. Most of survey questions were optional to allow participant to skip questions which they do not want to answer. Before implementation of the study, a preliminary pilot test for different sections of the survey was done among twenty potential participants. This was performed to check validity and clarity of the structured questions as well as to estimate the time needed to complete the survey. Accordingly, some questions about attitude and practice were restructured. Results of the pilot study were excluded from data analysis. All participants signed an informed consent before answering the survey.

### 2.1. Statistical Analysis

#### 2.1.1. Sample Size Calculation

We used the below equation to calculate the sample size for assuming a confidence interval level of 95%. The total sample size was determined to be 642 participants.

(1) $$\begin{array}{c} n=\frac{\left[\text{DEFF}*Np\left(1-p\right)\right]}{\left[\left(\left(d2/Z21\right)\right.-\left(\alpha /2\right)*\left(N-1\right)+p*\left(1-p\right)\right]}. \end{array}$$

Data were entered and validated using Microsoft Excel 2019, while the statistical analysis was done using the statistical package for the social sciences (SPSS, Windows version 22). All continuous quantitative data were presented in mean and standard deviation (SD), while categorical data were presented in frequencies and percentages. We used Student's *t*-test or ANOVA test to compare means and Chi-square test to compare frequencies. A *p* value of less than 0.05 was considered significant. Responses were collected in an online platform, and data were be analyzed to reveal the knowledge, applicability, and barriers of telemedicine in Egypt.

## 3. Results

**Figure 1:**
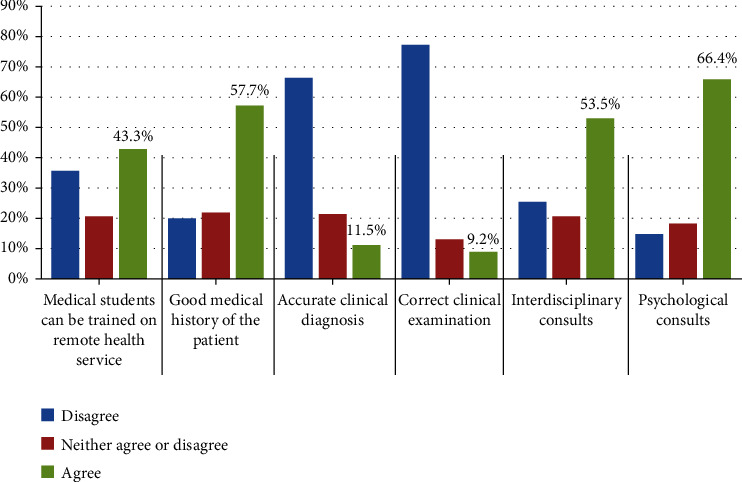
Extra services for remote medical services.

**Figure 2:**
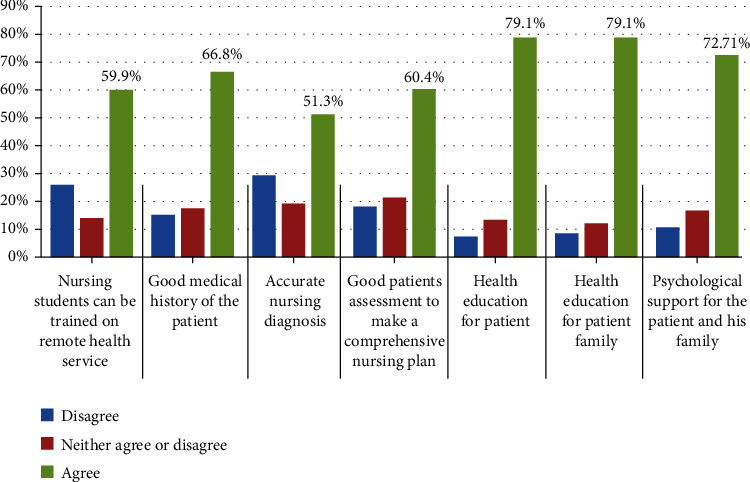
Extra services for remote nursing services.

Our survey included 642 healthcare providers from all over Egypt, 43.77% were females, with mean age 36 ± 9 years; their geographic distribution was 7.5% from frontiers governorates, 34.9% from urban governorates, 33.5% from rural governorates, and 24.1% from Upper Egypt governorates. Out of the 642 participants, 55.5% were physicians, 27.3% were nurses, 6.1% were technicians, 7.6% were administrative clerks, and 3.6% were medical directors. They were working in teaching hospitals or institutes (48.3%), general hospitals (29.5%), or private healthcare facilities (22.2%). Their mean duration of experience was 13.62 ± 8.56 years. Most of participating physicians (68%) specialized in general medicine or medical subspecialty, 17% were surgeons, 8% were specialized in laboratory medicine or radiology, and 7% were having other specialties. A total of 408 participants (64%) never used telemedicine in their practice while 234 (36%) used it. Older age and male gender were higher in the group who used telemedicine (35 ± 9 vs. 37 ± 9 and 52% vs. 64%); other variables including workplace and years of experience were statistically insignificant. Smartphones were used by at least 185 participants (79.1%) in the group who used telemedicine and smartphone applications were the favorable telemedicine service for 117 participants (50%) (Table 1). Regarding the impressions of healthcare workers about telemedicine necessity, most of participants (85.8%) were agreed to strongly agreed on its value in enhancing the contact between patients and their healthcare providers in emergency situations, while decreasing waiting lists and offering health services for remote deprived areas were agreed by 81.6% and 80.7% of the participants, respectively. About 89% of participants (*n* = 572) emphasized the need for clear policies and procedures for carrying out remote health service. Advantages and disadvantages of telemedicine according to opinion of participants are presented in Tables 2 and 3, respectively. Participants' opinion in the additional services that can be provided for physicians and nurses by telemedicine are presented in Figures 1 and 2, respectively.  Table 4 shows comparison of knowledge about remote health services among all participants (*n* = 642) according to their positions which include doctors (*n* = 365), nurses (*n* = 175), technicians (*n* = 39), administrative (*n* = 49), and directors (*n* = 23). Our data showed a statistically significant difference in telemedicine usage rates between the different groups, as directors were the highest group using telemedicine services (65.2%) while nurses were the lowest group (20.0%). There was a significant difference between groups regarding the technology devices used for remote calls (*p* = 0.02), at least one-third of directors reported they used all technology devices including mobiles, tablets, laptops, and computers. This percentage was higher comparing to other groups. However, most of the groups agreed that they use mobiles for their remote calls. Certain barriers that may face telemedicine such as availability of trained personnel and devices, knowing the basic technology and presence of technical support team were also screened in our study and presented in Table 5.

## 4. Discussion

It is clear that evolving technological achievements affect all levels of healthcare. Given the complexity of today's healthcare circumstances, successful telehealth adoption presents multidimensional and interprofessional challenges, depending mainly on human-related, social, and institutional factors [9]. In order to augment the utilization of these services, it was important to properly evaluate the situation based on user's opinions. So, we conducted this study aimed at assessing the knowledge, applicability, and barriers of telemedicine implementation among healthcare workers from different healthcare sectors and different Egyptian governorates [10]. In our study, most of the participants stated that they never used telemedicine before and this can pose great obstacles in the way of telemedicine implementation in general practice; consequently, different training courses should be performed to train HCWs on various telemedicine modalities. Our results were in accordance to the study conducted by Tom-Aba et al. during Ebola epidemic in 2015, which stated that doctors possess little knowledge concerning telemedicine [11]. However, this was in opposition to the study conducted by Woodward et al. which reported a positive doctor's attitude towards telemedicine utilization; the discrepancies in the results may be attributed to sociocultural effects owing to the heterogeneity of the study groups in terms of profession and residency [12]. The attitude of healthcare workers is the ultimate prerequisite for the successful incorporation of telemedicine services in healthcare facility systems, our study group impression about the importance of telemedicine implementation was marvelous, and they found it an excellent potentially cheap tool of delivering services to remote deprived areas; besides, it can save a lot of money, time, and efforts. And this was in agreement with the study conducted by Wernhart et al. in which the healthcare employees were very optimistic about the role of telemedicine in reduction of healthcare costs as well as data privacy and security [13]. On exploring the most prevailing mean of telemedicine application, participants indicated smartphone applications were the most convenient ones and were used by around 50% of the study group. Similarly, in a study conducted by Albarrak et al., 72% of participants prefer to interact via email and social media [14]. A closer look on the shortcomings of telemedicine in our study, telemedicine was not assumed to have a negative effect on the doctor patient relationship, and this was in contrary with what was proposed by Timmermans and Almeling that the advent of modern health technologies might badly affect the social bonds between doctors and patients [15]. This could be explained by the time gap between our study and Timmermans and Almeling study. Since 2009, health technologies and online communications have markedly improved and practiced in many healthcare facilities allowing more close communications between patients and their doctors. However, participants predicted that the implementation of telemedicine might reduce the quality of medical services offered to patients through increasing the probabilities of medical mistakes and lastly, the fear of inappropriate data protection might negatively influence the application of telemedicine as patient's privacy could be highly endangered. This result was in line with the Delphi survey conducted by Austrian health experts and other publications which stated that data privacy was the most serious problem encountered in telemedicine implementation [16].

In conclusion, despite the fact that telemedicine appears to be appealing and widely accepted by healthcare workers, yet, still its implementation is confronted by major obstacles including absence of trained personnel and devices, ignorance about the basic technology, lack of technical support, privacy and safety concerns, and legal and ethical issues. Thus, precise organizational guidelines have to clearly figure out the exact role and responsibilities of each healthcare worker to minimize the doubtfulness about telemedicine in order to facilitate its adoption [17].

## Figures and Tables

**Table 1:** Knowledge of telemedicine among the telemedicine users (*n* = 234).

Studied variable	No. (%)
*Which type of telemedicine do you know* ^∗^ *Participants could choose more than one*	
Video calls	72 (30.8)
Recorded calls	76 (32.5)
Remote observation	28 (12.0)
Mobile applications	135 (57.7)
I do not know any of them	29 (12.4)
*Type of technology devices used for remote calls*	
Mobile	153 (65.4)
Tablet	6 (2.6)
Laptop	14 (0.6)
Computer	6 (2.6)
Using some of the above	23 (9.8)
Using all of the above	32 (13.7)
*Telemedicine services used in your work* ^∗^ *Participants could choose more than one*	
Video call	55 (23.5)
Recorded calls	81 (34.6)
Remote observation	30 (12.8)
Mobile application	136 (58.1)
None of the above	29 (12.4)
*Favorable telemedicine service*	
Video call	42 18.0
Recorded calls	51 21.9
Remote observation	14 6.0
Mobile application	117 50.2
None of the above	9 3.9

**Table 2:** Remote health service advantages.

	*n* = 642				
	Strongly disagree, *N* (%)	Disagree, *N* (%)	Neither agree or disagree, *N* (%)	Agree, *N* (%)	Strongly agree, *N* (%)
Remote health service help for faster health service	13 (2.0)	31 (4.8)	145 (22.6)	364 (56.7)	89 (13.9)
Remote health is mandatory for patients care	12 (1.9)	45 (7.0)	133 (20.7)	373 (58.1)	79 (12.3)
Remote health service is important for remote-deprived areas	14 (2.2)	27 (4.2)	83 (12.9)	375 (58.4)	143 (22.3)
Carrying out remote health service needs clear policies and procedures	4 (0.6)	9 (1.4)	56 (8.7)	355 (55.3)	218 (34.0)
Remote health service saves effort	15 (2.3)	47 (7.3)	71 (11.1)	389 (60.6)	120 (18.7)
Remote health service saves money	17 (2.6)	32 (5.0)	109 (17.0)	377 (58.7)	107 (16.7)
Remote health service decrease waiting lists	15 (2.3)	33 (5.1)	70 (10.9)	403 (62.8)	121 (18.8)
Remote health service can help to provide patients with suitable information in emergency situations	12 (1.9)	25 (3.9)	54 (8.4)	378 (58.9)	173 (26.9)

**Table 3:** Remote health service disadvantages.

	*n* = 642				
	Strongly disagree, *N* (%)	Disagree, *N* (%)	Neither agree or disagree, *N* (%)	Agree, *N* (%)	Strongly agree, *N* (%)
Remote health service can have a negative effect on patient and healthcare provider	39 (6.1)	243 (37.9)	174 (27.1)	172 (26.8)	14 (2.2)
Remote health service can reduce medical service efficacy	26 (4.0)	171 (26.6)	146 (22.7)	256 (39.9)	43 (6.7)
Remote health service can cause psychological harm to the patient	41 (6.4)	313 (48.8)	177 (27.6)	99 (15.4)	12(1.9)
Remote health service can endanger patient privacy	41 (6.4)	288 (44.9)	144 (22.4)	155 (24.1)	14 (2.2)
Remote health service can cause disclosure of patient information to unauthorized persons	36(5.6)	213 (33.2)	163 (25.4)	214 (33.3)	16 (2.5)
Remote health service can increase service cost	55(8.6)	392 (61.1)	105 (16.4)	81 (12.6)	9 (1.4)
Remote health service can increase medical mistakes	24 (3.7)	139 (21.7)	156 (24.3)	260 (40.5)	63 (9.8)

**Table 4:** Comparison of knowledge about remote health service between healthcare workers.

Studied variable	Doctors (*N* = 356)	Nurses (*N* = 175)	Technicians (*N* = 39)	Administrative (*N* = 49)	Directors (*N* = 23)	*p* value
*Have you ever used telemedicine?*						
No	194(54.5)	140(80.0)	27(69.2)	39(79.6)	8(34.8)	<0.001
Yes	162 (45.5)	35 (20.0)	12(30.8)	10(20.4)	15(65.2)	

	*N* = 162	*N* = 35	*N* = 12	*N* = 10	*N* = 15	*p* value
*Which type of telemedicine do you know?* ^∗^ *Participants could choose more than one*						
Video calls	51 (31.5)	8 (22.8)	3 (25.0)	2 (20.0)	8 (53.3)	0.53
Recorded calls	60 (37.0)	5 (14.2)	2 (16.7)	4 (40.0)	5 (33.3)	
Remote observation	18 (11.1)	7 (20.0)	2 (16.7)	0 (0.0)	1 (6.7)	
Mobile applications	102 (62.9)	16 (45.7)	6 (50.0)	5 (50.0)	6 (40.0)	
I do not know any of them	18 (11.1)	3 (8.5)	1 (8.3)	2 (20.0)	3 (20.0)	
*Type of technology devices used for remote calls*						
Mobile	109 (67.3)	27 (77.1)	7 (58.3)	7 (70.0)	3 (20.0)	0.02
Tablet	5 (3.1)	0 (0.0)	0 (0.0)	0 (0.0)	1 (6.7)	
Laptop	10 (6.2)	1 (2.9)	0 (0.0)	1 (10.0)	2 (13.3)	
Computer	3 (1.9)	0 (0.0)	0 (0.0)	1 (10.0)	2 (13.3)	
Using some of the above	14 (8.6)	3 (8.6)	4 (33.3)	0 (0.0)	2 (13.3)	
Using all of the above	21 (13.0)	4 (11.4)	1 (8.3)	1 (10.0)	5 (33.3)	
*Telemedicine services used in your work* ^∗^ *Participants could choose more than one*						
Video call	35 (21.6)	8 (22.8)	2 (16.7)	2 (20.0)	7 (46.7)	0.39
Recorded calls	63 (38.9)	7 (20.0)	3 (25.0)	2 (20.0)	4 (26.7)	
Remote observation	16 (9.9)	8 (22.8)	3 (25.0)	0 0.0	2 (13.3)	
Mobile application	100 (61.7)	15 (42.8)	7 (58.3)	6 (60.0)	8 (53.3)	
None of the above	21 (12.9)	3 (8.6)	1 (8.3)	2 (20.0)	2 (13.3)	
*Favorable telemedicine service*						
Video call	28 (17.4)	6(17.1)	1 (8.30	1 (10.0)	6 (40.0)	0.2
Recorded calls	40 (24.8)	5 (14.3)	2 (16.7)	2 (20.0)	2 (13.3)	
Remote observation	6 (3.7)	5 (14.3)	2 (16.7)	1 (10.0)	0 (0.0)	
Mobile application	82 (50.9)	17 (48.6)	7 (58.3)	5 (50.0)	6 (40.0)	
None of the above	5 (3.1)	2 (5.7)	0 (0.0)	1 (10.0)	1 (6.7)	

**Table 5:** Remote health service barriers.

	Strongly agree to agree no (%)					
	Doctors (*N* = 35)	Nurses (*N* = 171)	Technicians (*N* = 17)	Administrative (*N* = 5)	Directors (*N* = 21)	*p* value Fisher's exact
Remote health service can be carried out easily in my specialty	157 44.4%	113 66.1%	13 76.5%	2 40.0%	9 42.9%	<0.001
My workplace has telemedicine facilities and trained personnel	117 33.1%	113 66.1%	6 35.3%	1 20.0%	10 47.6%	<0.001
I have enough time to be trained for telemedicine	162 45.8%	106 62.0%	9 52.9%	2 40.0%	10 47.6%	0.001
My work place has 24 hr technical support services	65 18.4%	78 45.6%	8 47.1%	1 20.0%	11 52.4%	<0.001
I know technology basics for remote health service	128 36.2%	89 52.0%	7 41.2%	3 60.0%	11 52.4%	0.003
I know how to protect data confidentiality	173 48.9%	124 72.5%	11 64.7%	4 80.0%	16 76.2%	<0.001

## Data Availability

The data used to support the findings of this study are included within the article (tables, figures, and supplementary table (available here).

## Supplementary Materials

Supplementary Materials Supplementary Table: impressions of healthcare workers about telemedicine.
