# Supplementary material for: Healthcare Providers' Perspective about the Use of Telemedicine in Egypt: A National Survey
Source: Int J Telemed Appl. 2022 Mar 12;2022:3811068. doi: 10.1155/2022/3811068 (PMC8934233; doi:10.1155/2022/3811068)
Supplement: Supplementary Materials — Supplementary Table: impressions of healthcare workers about telemedicine. [file 3811068.f1.docx]

Supplementary Table: Impressions of health-care workers about telemedicine

| Survey questions | Doctors  N=356 | Nurses  N=175 | Technicians  N=39 | Administrative  N= 49 | Directors  N=23 | p-value |
| --- | --- | --- | --- | --- | --- | --- |
| Have you ever used telemedicine?  Yes  No | 194(54.5)  162 (45.5) | 140 (80.0)  35 (20.0) | 27(69.2)  12(30.8) | 39(79.6)  10(20.4) | 8(34.8)  15(65.2) | <0.001 |
| Which type of Tele medicine do you know?  Video calls  Recorded calls  Remote observation  Mobile applications  I don’t know any of them | N=249  51 (20.5)  60 (24.1)  18 (7.2)  102 (40.9)  18 (7.2) | N=39  8 (20.5)  5 (12.8)  7 (17.9)  16 (41.0)  3 (7.7) | N=14  3 (21.4)  2 (14.3)  2 (14.3)  6 (42.9)  1 (7.1) | N=13  2 (15.4)  4 (30.8)  0 (0.0)  5 (38.5)  2 (15.4) | N=23  8(34.8)  5(21.8)  1(4.3)  6(26.1)  3 (13.0) | 0.53 |
| Remote health service help for faster health service | 232  65.2% | 140  80.0% | 28  71.8% | 39  79.6% | 14  60.9% | 0.05 |
| Remote health is mandatory for patients care | 229  64.3% | 141  80.6% | 32  82.1% | 37  75.5% | 13  56.5% | 0.01* |
| Remote health service is important for remote deprived areas | 278  78.1% | 147  84.0% | 33  84.6% | 42  85.7% | 18  78.3% | 0.83 |
| Remote health service offer for physicians and nurses rapid access to patients information | 297  83.4% | 151  86.3% | 30  76.9% | 44  89.8% | 18  78.3% | 0.28 |
| Carrying out remote health service needs clear policies and procedures | 320  89.9% | 156  89.1% | 35  89.7% | 43  87.8% | 19  82.6% | 0.02 |
| Remote health service saves effort | 258  72.5% | 157  89.7% | 33  84.6% | 45  91.8% | 16  69.6% | 0.003 |
| Remote health service saves money | 252  70.8% | 143  81.7% | 34  87.2% | 41  83.7% | 14  60.9% | 0.03 |
| Remote health service decrease waiting lists | 269  75.6% | 159  90.9% | 36  92.3% | 44  89.8% | 16  69.6% | <0.001 |
| Remote health service can enhance the contact between patients and their health care providers | 234  65.7% | 139  79.4% | 30  76.9% | 28  57.1% | 13  56.5% | 0.02 |
| Remote health service can help to provide patients with suitable information in emergency situations | 295  82.9% | 156  89.1% | 37  94.9% | 42  85.7% | 21  91.3% | 0.51 |
| Remote health service can have a negative effect on patient and health care provider | 122  34.3% | 43  24.6% | 5  12.8% | 9  18.4% | 7  30.4% | 0.03 |
| Remote health service can cause psychological harm to the patient | 65  18.3% | 37  21.1% | 3  7.7% | 4  8.2% | 2  8.7% | 0.17 |
| Remote health service can endanger patient privacy | 112  31.5% | 41  23.4% | 6  15.4% | 6  12.2% | 4  17.4% | 0.07 |
| Remote health service can cause disclosure of patient information to unauthorized persons | 144  40.4% | 56  32.0% | 11  28.2% | 11  22.4% | 8  34.8% | 0.05 |
| Remote health service can increase service cost | 45  12.6% | 33  18.9% | 4  10.3% | 4  8.2% | 4  17.4% | 0.69 |
